# Breast cancer recurrence and survival rates in patients who underwent breast‐conserving surgery under non‐mechanically ventilated anesthesia

**DOI:** 10.1002/cnr2.1643

**Published:** 2022-06-02

**Authors:** Ryungsa Kim, Ami Kawai, Megumi Wakisaka, Mika Shimoyama, Naomi Yasuda, Takanori Kin, Koji Arihiro

**Affiliations:** ^1^ Department of Breast Surgery Hiroshima Mark Clinic Hiroshima Japan; ^2^ Department of Breast Surgery Hiroshima City Hospital Hiroshima Japan; ^3^ Department of Anatomical Pathology Hiroshima University Hospital Hiroshima Japan

**Keywords:** breast cancer, breast‐conserving surgery, non‐mechanical ventilation anesthesia, recurrence, survival

## Abstract

**Background:**

Recurrence after primary treatment is an important obstacle to the curing of primary breast cancer. Less‐immunosuppressive anesthetic techniques, such as local anesthesia with lidocaine, intravenous anesthesia (IVA) with propofol, and/or sedation with midazolam under spontaneous breathing may reduce breast cancer recurrence compared with standard general anesthesia techniques such as IVA and inhalation anesthesia with opioids under mechanical ventilation.

**Aim:**

The aim of this study was to analyze the factors involved in breast cancer recurrence in patients who underwent breast‐conserving surgery (BCS) under non‐mechanically ventilated anesthesia.

**Methods:**

The study included 491 consecutive patients with stages 0–III breast cancer who underwent BCS/axillary lymph‐node management with local anesthesia and IVA and/or sedation under non‐mechanical ventilation between May 2008 and September 2021. Survival and recurrence were assessed by retrospective cohort analysis.

**Results:**

The median follow‐up period was 2565 days (range, 28–4834 days). The overall and breast cancer–specific survival rates were 92.9% and 95.6%, respectively. Twenty‐one deaths, of which 11 were breast cancer–related, occurred. Disease recurred in 29 (5.9%) patients, of whom 15 patients received neoadjuvant chemotherapy (NAC) and 14 patients received adjuvant therapy (chemotherapy in 12 cases). The surgical procedure performed, but not other clinicopathological factors [recurrence site, P stage, tumor subtype, and disease‐free interval (DFI)], differed between the NAC and adjuvant therapy groups. The DFI tended to be shorter in the NAC group than in the adjuvant therapy group. The pathological therapeutic effect grade after NAC was 1 in 12 patients and ≥2 in 3 patients.

**Conclusion:**

More than 50% (15/29) of patients with recurrence who underwent BCS were given NAC, but most patients did not respond to it. Similarly, adjuvant chemotherapy may not have contributed to the eradication of residual tumor cells after BCS. To reduce breast cancer recurrence in patients undergoing BCS, treatment strategies, especially for patients who do not respond to NAC or adjuvant chemotherapy, need to be developed. Non‐mechanical ventilation anesthesia may also affect the incidence of breast cancer recurrence.

## 1 INTRODUCTION

Over the past few decades, the development of systemic postsurgical therapies, such as anticancer drugs, molecular targeted drugs, and endocrine drugs, for a primary breast cancer has reduced breast cancer recurrence, and improved the patient survival rate.^1^, ^2^, ^3^ In addition, with the discovery of tumor subtypes and the introduction of multi‐gene assays, we can now select patients who will benefit from chemotherapy and individualize their treatment.^4^, ^5^ In contrast to the escalation of systemic therapies with neoadjuvant and adjuvant (e.g., dose‐dense) chemotherapies,^6^, ^7^ surgical treatment has been de‐escalated from mastectomy and axillary lymph‐node dissection (ALND) to breast‐conserving surgery (BCS) and sentinel lymph‐node biopsy (SLNB), even in locally advanced cases, with the use of neoadjuvant chemotherapy (NAC).^8^ This paradigm shift is based on the concept that breast cancer is a systemic disease, and that micrometastases are released from the primary tumor via lymphatic and vessel channels.^9^ In recent years, adjuvant molecularly targeted therapies with pertuzumab and trastuzumab for human epidermal growth factor 2 (HER‐2)‐positive breast cancer,^10^, ^11^ cyclin‐dependent kinases 4 and 6 (CDK4/6) with abemaciclib for hormone receptor (HR)‐positive/HER‐2–negative breast cancer,^12^ and programmed cell death 1 with pembrolizumab for triple‐negative (TN) breast cancer^13^ have been developed to prolong invasive disease‐free survival and to decrease morbidity associated with the primary breast cancer. Research indicates that these molecularly targeted agents can restore or enhance the drug sensitivity of some patients who do not respond well to anticancer or endocrine agents.^11^, ^12^, ^13^ However, the recurrence of breast cancer, such as with distant metastasis, is seen infrequently during follow‐up periods in clinical studies, meaning that the primary breast cancer has not been cured.

In general, breast cancer surgery is performed with standard forms of general anesthesia (GA), such as inhalation anesthesia with opioids or intravenous anesthesia (IVA) under mechanical ventilation. However, breast cancer surgeries such as BCS and SLNB can be performed under non‐mechanical ventilation with local anesthesia (LA; lidocaine) alone or in combination with propofol IVA and/or sedation with midazolam.^14^, ^15^, ^16^ Propofol has been found to be less suppressive of cell‐mediated immunity (CMI) than are inhalational anesthetics and opioids in mouse models and human samples,^17^, ^18^, ^19^ and local anesthetics such as lidocaine have been shown to inhibit breast cancer growth in vitro and in vivo.^20^ Furthermore, anesthesia with mechanical ventilation may induce cancer metastasis to the lungs and other parts of the body to a greater extent than does anesthesia with non‐mechanical ventilation, as suggested by data from mouse models.^21^, ^22^ Thus, LA and IVA with propofol and/or sedative agents without ventilation may play a role in reducing breast cancer recurrence compared to GA with ventilation. With this background, we retrospectively evaluated the survival and breast cancer recurrence rates of patients who underwent BCS and ALN management with LA and IVA with propofol and/or sedative agents under non‐mechanical ventilation in an outpatient setting.

## 2 METHODS

### 2.1 Patients

This retrospective study was performed with data from 491 women with a primary breast cancer (International Union for the Control of Cancer TNM stages 0–III)^23^ who underwent BCS under LA and IVA and/or sedation in an outpatient setting at the Hiroshima Mark Clinic, Hiroshima, Japan, between May 2008 and September 2021. Patients with stage IV disease were excluded. The decision to perform BCS was based on eligible patients' preference for outpatient over inpatient surgery; outpatient surgery was available, but never mandatory. The Ethics Committee of Hiroshima Mark Clinic approved this study (March 1, 2017), and all treatments were given with the patients' informed consent.

### 2.2 Anesthetic technique

LA with lidocaine, benzodiazepines (e.g., diazepam and midazolam) as sedatives, IVA with propofol, and pentazocine (an opioid receptor partial agonist) and pethidine (a synthetic opioid) as analgesics were used during the study period. Most frequently, each patient was given 30–80 ml 0.5% lidocaine in combination with IV propofol and/or midazolam. Preoperatively, 10 ml 0.5% lidocaine was injected locally into the posterior breast tumor space under ultrasound guidance when marking the tumor resection area; additional local injections were administered around the tumor before and during surgery. Pentazocine (15 mg) was administered as an analgesic. For intraoperative vital function monitoring, each patient was fitted with a biometric monitor to measure her pulse, electrocardiographic activity, blood pressure, respiratory rate, and oxygen saturation. In preparation for emergency mask ventilation and tracheal intubation, all patients were administered oxygen at a rate of 3–5 L/min by oxygen mask during surgery.

### 2.3 Surgical procedure

All patients underwent BCS with partial or quadrant breast resection plus SLNB and/or ALND; BCS was defined as partial breast resection with resection of the primary tumor and a 1–1.5 cm margin. Axillary SLNB was performed using indigo carmine and indocyanine green dyes. To evaluate SLN metastasis, one‐step nucleic acid amplification (OSNA),^24^ which identifies metastasis by measuring the cytokeratin 19 messenger RNA copy number in homogenized SLN samples, was performed intraoperatively. When metastases were detected by OSNA, limited ALND without drain insertion was performed in the level I area. Since August 2017, based on the results of the American College of Surgeons Oncology Group Z‐0011 study,^25^ ALND has not been performed for metastases to one or two lymph nodes. Patients who received NAC and had no clinical lymph‐node metastasis before chemotherapy underwent SLNB, and those with such metastasis confirmed by fine‐needle aspiration biopsy before chemotherapy underwent SLNB or ALND in the absence of clinical lymph‐node metastasis after chemotherapy.

### 2.4 Systemic and local therapies

NAC consisted of the combined administration of taxanes [e.g., paclitaxel (PTX), albumin‐bound nanoparticle paclitaxel (nab‐PTX), and docetaxel] and anthracyclines [e.g., 5‐fluorouracil/epirubicin/cyclophosphamide (FEC) and epirubicin/cyclophosphamide (EC)]. For patients with HER‐2‐positive breast cancer, trastuzumab alone or in combination with pertuzumab was combined with a taxane. Some patients were treated with dose‐dense therapy using EC and nab‐PTX. Therapeutic effects were assessed according to the histopathological criteria of the Japanese Breast Cancer Society.^26^, ^27^ Pathological therapeutic responses in intramammary lesions were graded as follows: grade 0, no or negligible change in cancer cells; grade 1a, mild changes in cancer cells, regardless of area, or marked changes in <1/3 of cancer cells; grade 1b, marked changes in ≥1/3 but <2/3 of cancer cells; grade 2a, marked changes in ≥2/3 of cancer cells; grade 2b, disappearance of almost all cancer cells; and grade 3, apparent disappearance of all cancer cells. Therapeutic efficacy was classified according to the involvement of the breast ducts and/or ALNs.

The patients received adjuvant therapy according to tumor subtype and pathological findings for the primary tumor. Some treatments were determined by Oncotype‐DX (Genomic Health, Redwood City, CA, USA) test results at the patients' wishes because this test was not covered by health insurance in Japan. Lymph node‐positive patients and high‐risk node‐negative patients received adjuvant chemotherapy with an anthracycline and a taxane. Other node‐negative patients were treated with docetaxel/cyclophosphamide, PTX, or FEC. Fifty‐one patients who did not want to receive standard chemotherapy because of alopecia and intermediate risk were treated with the oral fluoropyrimidine anticancer agents tegafur‐uracil (UFT) and tegafur/gimeracil/oteracil (TS‐1). Patients with positive HRs were given endocrine therapy, with extension of this therapy depending on their risk level.

Radiation therapy (RT) to the rest of the breast was administered at a partner hospital using standard doses, 3 weeks (hypofractionated doses: 40.5 Gy/15 Fr) or 4 weeks (conventional dose: 50 Gy/25 Fr) after surgery or adjuvant chemotherapy. The actual radiation dose was adjusted according to the body surface area. Cases with resection margins ≤5 mm or age ≤ 50 years were given RT boosters to the tumor bed, with additional boosters (10.8 Gy/4 Fr or 10 Gy/5 Fr) administered as needed; in cases with ALN metastases before and after NAC, additional irradiation of the regional lymph nodes was included after surgical treatment. Neoadjuvant therapy (chemotherapy or endocrine therapy) was administered for 6 months before surgery to patients with tumors larger than 4 cm or ALN metastasis. All patients with stage III cancer and some patients with stage II cancer received NAC preoperatively.

### 2.5 Statistical analyses

All data were analyzed using Statcel 4 software (OMS Publishing Inc., Tokyo, Japan). Continuous and independent variables [e.g., P stage and disease‐free interval (DFI)] were compared between groups using the Mann–Whitney test. Categorical variables (e.g., recurrence site, tumor subtype, and surgical procedure) were analyzed using the chi‐squared test. Cumulative overall survival (OS) and breast cancer‐specific survival (BCSS) rates were calculated using the Kaplan–Meier method, and compared among groups using the log‐rank test. *P* values <0.05 were considered to be significant.

## 3 RESULTS

### 3.1 Patients' clinicopathological characteristics

Of 797 patients who were eligible for BCS with axillary management, 491 requested outpatient surgery and 306 requested inpatient surgery and were referred to the hospital of their choice. The 491 patients included in this study had a median age of 50 years (ranges, 27–91 years), and the predominant clinical stage at the time of diagnosis was I (54.1%), followed by II (35.8%). The main pathological tumor size classification was T1 (64.3%), followed by T2 (17.3%). Pathological axillary lymph‐node metastasis was found in 113 (22.9%) patients. The most common tumor nuclear grade was 3 (49.2%), followed by 2 (37.0%). HR‐positive and HER‐2–negative cancer was identified in 349 (71.0%) patients; 59 (11.9%) patients had HER‐2‐positive cancer and 21 (4.2%) patients had TN cancer. The most common surgery performed was partial breast resection with SLNB (76.7%), followed by ALND and initial ALND. A total of 82 (16.7%) patients received NAC and 239 (48.6%) patients received adjuvant chemotherapy. Postoperative RT was administered to 447 (91.0%) patients, and RT boosters were administered in 254 (51.7%) of these cases. The clinicopathological characteristics of the patients are shown in Table 1.

**TABLE 1:** Clinicopathological characteristics of 491 patients who underwent breast‐conserving surgery

Clinicopathological characteristic	*n* (%) or median (range)
Age, years	50 (27–91)
Stage at diagnosis	
0	22 (4.4)
I	266 (54.1)
II	176 (35.8)
III	27 (5.4)
Pathological tumor size	
T0	11 (2.2)
Tis	74 (15.0)
T1	316 (64.3)
T2	85 (17.3)
T3	5 (1.0)
Pathological nodal status	
N0	376 (76.5)
N1	105 (21.3)
N2	8 (1.6)
Unknown	2 (0.4)
Tumor histology	
IDC	350 (71.2)
ILC	10 (2.0)
Other IDC	47 (9.5)
NIDC	73 (14.8)
Nuclear grade	
I	53 (10.7)
II	182 (37.0)
III	242 (49.2)
Unknown	3 (0.6)
Tumor subtype	
HR+/HER‐2–	349 (71.0)
HR+/HER‐2+	49 (9.9)
HR–/HER‐2+	10 (2.0)
Triple negative	21 (4.2)
Surgery type	
Bp/SLNB	377 (76.7)
Bp/SLNB/ALND	31 (6.3)
Bp/ALND	58 (11.8)
Other	25 (5.0)
Neoadjuvant chemotherapy	82 (16.7)
Adjuvant chemotherapy	239 (48.6)
Postoperative radiotherapy^a^	447 (91.0)
Conventional	261 (53.1)
Hypofractionated	185 (37.6)
Dose unknown	1 (0.2)
Boosted	254 (51.7)

Abbreviations: IDC, invasive ductal carcinoma; ILC, invasive lobular carcinoma; NIDC, noninvasive ductal carcinoma; HR, hormone receptor; HER‐2, human epidermal growth factor receptor 2; Bp, partial breast resection; SLNB, sentinel lymph‐node biopsy; ALND, axillary lymph‐node dissection.

^a^ Unknown for seven cases.

### 3.2 Anesthetic techniques and surgical complications

The anesthetic techniques used for BCS did not involve ventilation anesthesia; they were based on LA with lidocaine and are summarized in Table 2. The most frequently used anesthetic technique was lidocaine/propofol/midazolam/pentazocine or pethidine [*n* = 223 (45.4%)], followed by lidocaine/diazepam/midazolam/pentazocine or pethidine [*n* = 108 (21.9%)] and lidocaine/propofol/pentazocine or pethidine [*n* = 96 (19.5%)]. In total, 250 (50.9%) patients received pethidine and 241 (49.0%) patients received pentazocine as analgesia. Other techniques included the use of lidocaine alone or in combination with diazepam or midazolam and pentazocine or pethidine.

**TABLE 2:** Anesthetic techniques

Anesthesia/sedation/analgesia	*n* (%)
Lidocaine/propofol/midazolam/pentazocine or pethidine	223 (45.4)
Lidocaine/diazepam/midazolam/pentazocine or pethidine	108 (21.9)
Lidocaine/propofol/pentazocine or pethidine	96 (19.5)
Others	64 (13.0)

Regarding surgical complications, wound infection was observed in 16 (3.0%) patients, and postoperative hematoma was observed in 18 (3.6%) patients, 5 of whom underwent reoperation 5–7 days after surgery to stop bleeding at the resected site. Axillary lymphoceles were observed in 33 (7.2%) of the 84 patients undergoing limited ALND without axillary drainage; these lymphoceles disappeared after several aspirations, and no case required continued management after surgery.

### 3.3 Survival and breast cancer recurrence

**FIGURE 1:**
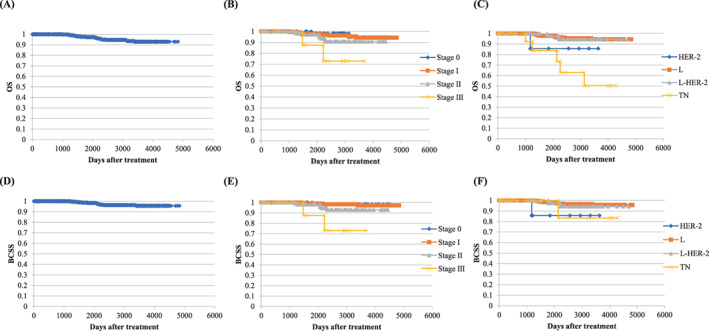
Cumulative overall survival (A), overall survival by pathological stage (B) and tumor subtype (C), and breast cancer–specific survival overall (D) and by pathological stage (E) and tumor subtype (F) for 491 patients with breast cancer who underwent breast‐conserving surgery in an outpatient setting with local and intravenous anesthesia and/or sedation. “Days after treatment” refers to the number of days after surgery or systemic treatment. OS, overall survival; HER‐2, human epidermal growth factor receptor 2; L, luminal; TN, triple negative BCSS, breast cancer‐specific survival

Over a median follow‐up period of 2565 days (ranges, 28–4834 days), the cumulative OS rate for the total cohort was 92.9%; OS rates for stages 0–III disease were 94.6%, 94.3%, 90.8%, and 72.9%, respectively (*P* = 0.0239). OS rates for luminal, luminal HER‐2, HER‐2, and TN breast cancers were 94.5%, 94.3%, 85.7%, and 50.3%, respectively (*P* = 0.0007; Figure 1). The BCSS rate for the total cohort was 95.6%; BCSS rates for pStages 0–III disease were 98.2%, 97.2%, 92.9%, and 72.9%, respectively (*P* = 0.0010). BCSS rates for L, L‐HER2, HER2, and TN breast cancers were 95.7%, 94.3%, 85.7%, and 83.3%, respectively (*P* = 0.1617; Figure 1).

Of the 21 deaths that occurred, 11 were breast cancer–related and 10 were not. Disease recurrence was observed in 29 (5.9%) patients. Of these, 15 patients received NAC and 14 patients received adjuvant therapy. Most clinicopathological characteristics, such as the recurrence site, P stage, tumor subtype, and DFI, did not differ significantly between groups (Table 3). One exception was the surgical procedure performed; most (14 of 15) patients in the NAC group underwent partial or quadrant breast resection with ALND, whereas most (9 of 14) of those in the adjuvant therapy group underwent partial breast resection with SLNB. Another exception was RT booster administration after surgical treatment, which was more common among patients who received NAC than among those who received adjuvant chemotherapy. The DFI tended to be shorter in the NAC group than in the adjuvant therapy group; the rates of recurrence within 2 years after surgical treatment were 53.3% (8 of 15 patients) and 28.5% (4 of 14 patients), respectively.

**TABLE 3:** Clinicopathological characteristics of 29 breast cancer recurrences in patients receiving neoadjuvant chemotherapy and adjuvant therapy before and after breast‐conserving surgery

Clinicopathological factor	No. of patients		*P*
	NAC (*n* = 15)	Adjuvant therapy (*n* = 14)	
Recurrence site			0.7830^a^
Local	3	5	
Regional	2	1	
Liver	2	2	
Lung	1	1	
Bone	0	1	
Brain	1	0	
Multiple	6	4	
P stage			0.6000^b^
0	1	2	
I	6	6	
II	6	4	
III	2	2	
Subtype			0.1887^a^
Luminal	10	9 (7 chemo)	
Luminal/HER‐2	1	3 (3 chemo)	
HER‐2	1	0	
Triple negative	3	0	
Surgical procedure			0.00001^a^
Bp	0	2	
Bp + SLNB	1	9	
Bp + SLNB+ALND	0	3	
Bp/Bq + ALND	14	0	
DFI			0.2474^b^
≤ 2 years	8	4	
> 2 to ≤5 years	5	7	
> 5 years	2	3	
RT			
Conventional	12	10	0.3087^a^
Hypofractionated	3	2	
None	0	2	
Booster+	11	4	0.0376^a^
Booster–	4	8	

Abbreviations: NAC, neoadjuvant chemotherapy; HER‐2, human epidermal growth factor receptor 2; Bp, partial breast resection; SLNB, sentinel lymph‐node biopsy; ALND, axillary lymph‐node dissection; Bq, quadrant breast resection; DFI, disease‐free interval; RT, radiotherapy.

^a^ Chi‐squared test.

^b^ Mann–Whitney test.

The clinicopathological characteristics of the patients in the NAC group are shown in Table 4. A total of 10 of these 15 patients had luminal type tumors, 2 were HER‐2‐positive, and 3 had TN breast cancer. Pathological therapeutic responses were grade 1 in 12 patients, grade 2 in 2 patients, and grade 3 in 1 patient. A total of 8 of the 12 patients with grade 1 responses had luminal breast cancer. Thus, most locally advanced luminal breast cancers in this sample extended to the regional lymph nodes and did not respond to NAC with anthracyclines and taxanes. Recurrence to lymph nodes in the axillary, supraclavicular, and internal mammary regions was detected in 7 of the 15 patients, suggesting that tumor cells remaining after NAC did not respond to postoperative RT (including booster) administration to the breast and regional lymph nodes. Furthermore, residual disease was not eradicated by adjuvant endocrine therapy or oral fluoropyrimidine anticancer agents such as UFT, TS‐1, and capecitabine. Similarly, three cases of grade 1 TN breast cancer did not respond to NAC, and adjuvant therapy with oral fluoropyrimidine did not prevent recurrence.

**TABLE 4:** Clinicopathological characteristics of 15 patients with recurrent breast cancer who underwent breast‐conserving surgery after neoadjuvant chemotherapy under non‐mechanical ventilation anesthesia

Pt no.	Age at diagnosis (years)	Tumor subtype	TNM, C stage	Regimen	Surgical procedure	Pathological therapeutic response (grade)	Postoperative RT	Postoperative adjuvant therapy	Recurrence site	Disease‐free interval	Outcome/ remarks
1	40	Luminal	T2N1M0, stage IIB	DTX/FEC	Bp + SLNB	1a	60 Gy/30 Fr, B^+^/R^−^	TAM + LH‐RHa/UFT	Local	9Y3M	Alive
2	35	L–HER2	T2N1M0, stage IIB	DTX + Tz/FEC	Bp + ALND	3	50 Gy/25 Fr, B^−^/R^−^	TAM/LH‐RHa/Tz	Brain	1Y	Dead
3	63	Luminal	T1cN2M0, stage IIIA	PTX/FEC	Bp + ALND	2b	50 Gy/25 Fr, B^−^/R^−^	ANZ	IMLNs/bone	6Y3M	Dead
4	44	TN	T2N1M0, stage IIB	Nab‐PTX/FEC	Bp + ALND	1a	40.5 Gy/15 Fr, B^−^/ R^−^	TS‐1	Ax/Sc LNs	1Y3M	Dead
5	52	Luminal	T2N3M0, stage IIIC	DTX/FEC	Bp + ALND	2a	60.4 Gy/33 Fr, B^+^/IM + Sc	LEZ/UFT	Lung/hilar LNs	3Y	Dead
6	67	HER2	T2N3M0, stage IIIC	Nab‐PTX + Tz/FEC	Bp + ALND	1a	60 Gy/30 Fr, B^+^/Sc	Tz/TS‐1	Ax/Sc/lung hilar LNs	1Y2M	Dead
7	44	Luminal	T2N1M0, stage IIB	Nab‐PTX/FEC	Bq + ALND	1b	60 Gy/30 Fr, B^+^/Sc	TAM + LH‐RHa/TS‐1	Liver/retropancreatic and para‐aortal LNs	2Y	Dead
8	43	Luminal	T2N1M0, stage IIB	Nab‐PTX/FEC	Bp + ALND	1b	60 Gy/30 Fr, B^+^/Sc	TAM(TOR) + LH‐RHa /UFT	Liver	1Y7 M	Dead
9	51	Luminal	T2N3M0, stage IIIC	Nab‐PTX/FEC	Bp + ALND	1b	60 Gy/30 Fr, B^+^/IM + Sc	LEZ/TS‐1	Sc/mediastinal LNs/lung/brain	1Y6M	Dead/3 FEC cycles
10	59	Luminal	T2N3M0, stage IIIC	Nab‐PTX/FEC	Bp + ALND	1a	60 Gy/30 Fr, B^+^/IM + Sc	LEZ/UFT	Ax/Sc LNs	2Y8M	Dead/1 FEC cycle
11	62	Luminal	T2N3M0, stage IIIC	FEC/nab‐PTX	Bp + ALND	1a	60 Gy/30 Fr, B^+^/IM	EXE/TS‐1	Liver	4Y1M	Dead
12	46	Luminal	T2N1M0, stage IIB	EC/DTX	Bp + ALND	1a	60 Gy/30 Fr, B^+^/Sc	TAM/TS‐1	Local	7 M	Unknown
13	52	TN	T2N0M0, stage IIA	ddEC/nab‐PTX	Bp + ALND	1a	51.3 Gy/19 Fr, B^+^/Sc	Capecitabine	Local	2Y3M	Alive
14	40	Luminal	T2N3M0, stage IIIC	ddEC/ddnab‐PTX	Bp + ALND	1b	40.5 Gy/15 Fr, B^−^/IM + Sc	LH‐RHa + TAM/Capecitabine	Ax LN/lung	2Y5M	Alive
15	61	TN	T2N3M0, stage IIIC	EC/PTX	Bp + ALND	1a	60 Gy/30 Fr, B^+^/Sc	Capecitabine/CPA	Local/Sc/Ax/mediastinal/lung hilar/para‐aortal LNs/liver	11 M	Alive

Abbreviations: Pt, patient; no., number; RT, radiation therapy; DTX, docetaxel; FEC, 5‐FU/epirubicin/cyclophosphamide; Bp, partial breast resection; SLNB, sentinel lymph‐node biopsy; B, booster; R, regional; TAM, tamoxifen; LH‐RHa, luteinizing hormone‐releasing hormone agonist; UFT, tegafur/uracil; Y, years; M, months; L–HER2, luminal–human epidermal growth factor receptor 2; Tz, trastuzumab; ALND, axillary lymph‐node dissection; PTX, paclitaxel; ANZ, anastrozole; IMLN, internal mammary lymph node; TN, triple negative; Nab‐PTX, nanoparticle albumin–bound paclitaxel; TS‐1, tegafur/gimeracil/oteracil; Ax, axillary; Sc, supraclavicular; LN, lymph node; IM, internal mammary; LEZ, letrozole; Bq, breast quadrantectomy; TOR, toremifene; EXE, exemestane; EC, epirubicin/cyclophosphamide; ddEC, dose‐dense EC; ddnab‐PTX, dose‐dense nab‐PTX; CPA, cyclophosphamide.

Of the 14 cases of recurrence treated adjuvantly (e.g., with anticancer drugs and endocrine therapy), 7 recurred at 2–5 years after surgery (Table 5). A total of 12 of these 14 patients (2 of whom had ductal carcinoma in situ; 9 luminal, 3 luminal/HER‐2) received postoperative adjuvant chemotherapy. Thus, postoperative adjuvant chemotherapy and endocrine therapy did not effectively eradicate residual tumor cells.

**TABLE 5:** Clinicopathological characteristics of 14 patients with recurrent breast cancer who underwent breast‐conserving surgery with adjuvant therapy under non‐mechanical ventilation anesthesia

Pt no.	Age at diagnosis (years)	Histology	Tumor subtype	TNM, C stage	Surgical procedure	Chemotherapy regimen	Postoperative RT	Postoperative adjuvant therapy	Recurrence site	Disease‐free interval	Outcome/remarks
1	27	DCIS	ND	T1cN0M0, stage I	Bp	None	60 Gy/30 Fr, B^+^/R^−^	None	Local	2Y11 M	Alive
2	68	IDC	Luminal	T1cN0M0, stage I	Bp	None	None	ANZ	Local	9Y2M	Alive
3	34	IDC	Luminal	T1cN0M0, stage I	Bp + SLNB	DTX/FEC	50 Gy/25 Fr, B^−^/R^−^	TAM + LH‐RHa	Liver/hilar LNs	7Y7 M	Alive
4	52	IDC	Luminal	T1cN0M0, stage I	Bp + SLNB	FEC	50 Gy/25 Fr, B^−^/R^−^	ANZ	Bone/Ax/Sc/mediastinal/lung/pleura	6Y1M	Unknown
5	46	IDC	Luminal	T2N0M0, stage IIA	Bp + SLNB	DTX/FEC	50 Gy/25 Fr, B^−^/R^−^	TAM + LH‐RHa	Brain/bone	2Y	Dead
6	58	IDC	Luminal	T1cN0M0, stage I	Bp + SLNB+ALND	PTX/FEC	None	LEZ	Liver	3Y9M	Unknown
7	51	IDC	L‐HER2	T1cN0M0, stage I	Bp + SLNB	PTX + Tz/FEC	50 Gy/25 Fr, B^−^/R^−^	Tz/LEZ	Local/mediastinal, hilar LNs/lung	3Y7 M	Alive
8	52	IDC	L‐HER2	TisN0M0, stage 0	Bp + SLNB	TC + Tz	50 Gy/25 Fr, B^−^/R^−^	Tz/LEZ	Local	4Y8M	Alive
9	35	IMPC	Luminal	T2N0M0, stage IIA	Bp + SLNB	DTX/FEC	51.3 Gy/19 Fr, B^−^/R^−^	TAM + LH‐RHa	Lung	3Y3M	Alive
10	48	IDC	L‐HER2	T1cN0M0, stage I	Bp + SLNB+ALND	FEC/nab‐PTX + Tz	60 Gy/30 Fr, B^+^/Sc	Tz/LEZ	Local	1Y1M	Dead
11	62	IDC	Luminal	T2N0M0, stage IIA	Bp + SLNB	TC	50 Gy/25 Fr, B^−^/R^−^	ANZ	Bone	1Y	Dead
12	47	DCIS	ND	TisN0M0, stage 0	Bp + SLNB	None	50 Gy/25 Fr, B^+^/R^−^	TAM	Local	4Y	Alive
13	39	IDC	Luminal	T2N0M0, stage IIA	Bp + SLNB+ALND	DTX/FEC	60 Gy/30 Fr, B^+^/Sc	TAM + LH‐RHa /TS‐1	Liver/lung	4Y4M	Alive
14	82	IDC	Luminal	T1cN0M0, stage I	Bp + SLNB	None	40.5 Gy/15 Fr, B^−^/R^−^	LEZ	Ax/Sc LNs	1Y	Alive

Abbreviations: Pt, patient; no., number; RT, radiation therapy; DCIS, ductal carcinoma in situ; ND, not determined; Bp, partial breast resection; B, boost; R, regional; Y, years; M, months; IDC, invasive ductal carcinoma; ANZ, anastrozole; DTX, docetaxel; FEC, 5‐FU/epirubicin/cyclophosphamide; SLNB, sentinel lymph‐node biopsy; TAM, tamoxifen; LH‐RHa, luteinizing hormone‐releasing hormone agonist; LN, lymph node; Ax, axillary; Sc, supraclavicular; PTX, paclitaxel; ALND, axillary lymph‐node dissection; LEZ, letrozole; L–HER2, luminal–human epidermal growth factor receptor 2; Tz, trastuzumab; TC, docetaxel/cyclophosphamide; IMPC, invasive micropapillary carcinoma; nab‐PTX, nanoparticle albumin–bound paclitaxel; TS‐1, tegafur/gimeracil/oteracil.

## 4 DISCUSSION

In this study, we retrospectively analyzed the survival and breast cancer recurrence rates in a sample of 491 patients who underwent BCS and ALN management with LA (lidocaine) and IVA and/or sedation under non‐mechanical ventilation. Over a median follow‐up period of more than 7 years, the OS and BCSS rates exceeded 90%. The breast cancer recurrence rate (5.9%) was lower than previously reported rates of 10–20%,^28^, ^29^ possibly due to the maintenance of host immunity through the use of less‐immunosuppressive anesthetic techniques. The use of LA for breast cancer surgery has been shown to provoke a lesser lymphoproliferative response than does the use of GA.^14^ Excellent survival rates (OS, BCSS, and according to the P stage and tumor subtype) with the use of this approach have been reported.^15^, ^16^ However, even with the use of non‐mechanically ventilated anesthesia, breast cancer recurrence (i.e., local or distant metastasis) occurs infrequently in patents who have received NAC or adjuvant therapy before and after surgical treatment. Thus, primary treatment cannot be considered to cure the primary breast cancer.

The DFI tended to be shorter in the NAC group than in the adjuvant therapy group, and this tendency was reflected in the lesser pathological therapeutic response to NAC in patients with recurrence, in this study. Considerable residual disease after NAC may not be eradicated or suppressed by additional adjuvant oral fluoropyrimidine or endocrine therapy; it may be resistant to oral anticancer agents, anthracyclines, and taxanes. Additional adjuvant chemotherapy with capecitabine for residual disease after NAC has been shown to improve disease‐free survival and OS, especially among patients with TN breast cancer.^30^ Patients with no recurrence after NAC and adjuvant therapy may respond to additional adjuvant therapy, even if that response is below grade 2, suggesting that therapeutic effects on residual tumor cells depend on their sensitivity to oral fluoropyrimidines and endocrine therapy.

The treatment of patients who have not responded to NAC and the overcoming of drug resistance in non‐responders are important issues that need to be resolved. One possible reason for a low response to NAC is that anticancer drugs do not activate CMIs such as natural killer (NK) cells and cytotoxic T lymphocytes. In patients with post‐NAC responses of grade 2 or higher, the activation of NK cells and CD8 cells in tumors, the down‐regulation of CTLA‐4 in regulatory T cells as a local immune response, and increases in peripheral NK cells and T lymphocytes as a systemic immune response, have been observed.^31^, ^32^ These NAC‐induced immune activations are thought to involve immunogenic cell death by anthracyclines and immunogenic modulation by taxanes, which can reactivate antitumor immunity by modulating tumor‐infiltrating lymphocytes in the immunosuppressive tumor microenvironment.^33^, ^34^, ^35^ Immunogenic cell death and immunogenic modulation by adjuvant chemotherapy may also explain the eradication of residual tumor cells after surgical treatment, resulting in the curing of the primary breast cancer, in responders.^36^ The immunomodulatory effects of anticancer drugs have been reported not only for anthracyclines and taxanes, but also for oral fluoropyrimidines such as capecitabine.^37^, ^38^, ^39^ Thus, successful immune activation by anticancer agents may eradicate residual tumor cells after surgical treatment and lead to a cure, but which factors are need for such activation remains unclear.

Residual tumor cells that do not respond to NAC or adjuvant therapy after surgical treatment may be cancer stem cells^40^ or dormant‐disseminated tumor cells (DTCs)^41^ that are resistant to anticancer drugs and RT and evade host defense immunity. Breast cancer stem cells (BCSCs) can self‐renew and differentiate to generate non‐neoplastic tumor cells that form tumor masses and promote tumor progression and metastasis.^42^ Dormant DTCs, some of which may be consistent with BCSCs, can survive as quiescent cells for long periods of time in the bone marrow and pre‐metastatic pulmonary niches.^42^, ^43^ These cells are thought to be awakened by inflammatory responses and the activation of signaling pathways in the tumor microenvironment, leading to tumor regrowth and the formation of metastatic lesions.^43^ Additional research is needed to guide the development of targeted strategies for BCSC and dormant DTC elimination, contributing to the curing of the primary breast cancer.

Although the stress response to BCS and ALN management is low, the anesthetic technique is an important factor affecting host immune defense in the perioperative period. The relationship between the anesthetic technique and recurrence is controversial in the field of oncological surgery, but randomized controlled trials (RCTs) have not provided sufficient evidence to date that the anesthetic technique is associated with the recurrence rate or long‐term outcomes in patients undergoing breast cancer surgery.^44^, ^45^ In addition, clinical studies have provided no evidence that the use of mechanical ventilation during breast cancer surgery increases breast cancer recurrence relative to the use of non‐mechanical ventilation. In a preclinical study, however, mechanical ventilation during mastectomy under GA in mice implanted with breast cancer cell lines significantly increased the number of circulating breast cancer cells remaining in lung micrometastases and the incidence of postoperative lung metastasis.^21^ Moreover, the paracrine secretion of pro‐inflammatory cytokines may induce metastasis to organs other than the lung.^22^ Although no evidence currently supports an effect of the anesthesia technique on the long‐term prognoses of patients with breast cancer^46^; the use of less‐immunosuppressive anesthesia under spontaneous breathing for BCS may reduce breast cancer recurrence compared with the use of standard GA under mechanical ventilation, but no such direct comparative analysis was performed in this study, constituting a study limitation. RCTs comparing the outcomes achieved with the use of less‐immunosuppressive anesthetic techniques without mechanical ventilation with those achieved with the use of standard GA with mechanical ventilation for BCS in patients with breast cancer are needed.

Even with the use of less‐immunosuppressive anesthesia, NAC and adjuvant therapy are not effective, and the subsequent immune activation is not elicited to eradicate residual tumor cells, making breast cancer recurrence inevitable, in certain cases. Some drug‐resistant residual tumors in patients with recurrence may be overcome by the combined administration of CDK4/6 inhibitors, immune checkpoint inhibitors, and other molecular targeted therapies. To reduce breast cancer recurrence, predictive markers of the effective induction of antitumor immune responses need to be identified and individualized treatment strategies for non‐responsive tumors need to be developed.

## 5 CONCLUSION

Although BCS and ALN management provoke less surgical stress than do other procedures for breast cancer, the use of less‐immunosuppressive anesthesia under non‐mechanical ventilation may contribute to the reduction of breast cancer recurrence. The molecular mechanism underlying the activation of antitumor immunity via the immunomodulatory effects of anticancer drugs needs to be elucidated. The development of therapeutic strategies for drug‐resistant tumors and the eradication of residual tumor cells by enhancing host defense immunity would be a promising breakthrough leading to the elimination of breast cancer recurrence.

## AUTHOR CONTRIBUTIONS

**Ryungsa Kim**: Conceptualization (equal); data curation (equal); investigation (equal); methodology (equal); project administration (equal); resources (equal); visualization (equal); writing – original draft (equal); writing – review and editing (equal). **Ami Kawai**: Data curation (equal); formal analysis (equal); investigation (equal); resources (equal); software (equal); visualization (equal); writing – review and editing (equal). **Megumi Wakisaka**: Resources (equal). **Mika Shimoyama**: Resources (equal). **Naomi Yasuda**: Resources (equal). **Takanori Kin**: Resources (equal). **Koji Arihiro**: Resources (equal).

## FUNDING

No funding was received for this study.

## CONFLICT OF INTEREST

The authors declare that they have no conflict of interest.

## ETHICS STATEMENT

This study was performed in accordance with the Declaration of Helsinki. The Ethics Committee of Hiroshima Mark Clinic (March 1, 2017) approved the study. All treatments were given with the patients' informed consent.

## Data Availability

Data sharing is not applicable to this article as no new data were created or analyzed in this study.

## References

[1] Caparica R , Brandão M , Piccart M . Systemic treatment of patients with early breast cancer: recent updates and state of the art. Breast. 2019;48(Suppl 1):S7‐S20. 31839166 10.1016/S0960-9776(19)31115-4

[2] Early Breast Cancer Trialists' Collaborative Group (EBCTCG) , Peto R , Davies C , Godwin J , et al. Comparisons between different polychemotherapy regimens for early breast cancer: meta‐analyses of long‐term outcome among 100,000 women in 123 randomised trials. Lancet. 2012;379(9814):432‐444. 22152853 10.1016/S0140-6736(11)61625-5 PMC3273723

[3] Early Breast Cancer Trialists' Collaborative Group (EBCTCG) . Aromatase inhibitors versus tamoxifen in early breast cancer: patient‐level meta‐analysis of the randomised trials. Lancet. 2015;386(10001):1341‐1352. 26211827 10.1016/S0140-6736(15)61074-1

[4] Perou CM , Sørlie T , Eisen MB , et al. Molecular portraits of human breast tumours. Nature. 2000;406(6797):747‐752. 10963602 10.1038/35021093

[5] Sparano JA , Gray RJ , Makower DF , et al. Adjuvant chemotherapy guided by a 21‐gene expression assay in breast cancer. N Engl J Med. 2018;379(2):111‐121. 29860917 10.1056/NEJMoa1804710 PMC6172658

[6] Early Breast Cancer Trialists' Collaborative Group (EBCTCG) . Long‐term outcomes for neoadjuvant versus adjuvant chemotherapy in early breast cancer: meta‐analysis of individual patient data from ten randomised trials. Lancet Oncol. 2018;19(1):27‐39. 29242041 10.1016/S1470-2045(17)30777-5 PMC5757427

[7] Early Breast Cancer Trialists' Collaborative Group (EBCTCG) . Increasing the dose intensity of chemotherapy by more frequent administration or sequential scheduling: a patient‐level meta‐analysis of 37 298 women with early breast cancer in 26 randomised trials. Lancet. 2019;393(10179):1440‐1452. 30739743 10.1016/S0140-6736(18)33137-4 PMC6451189

[8] Morrow M . De‐escalating and escalating surgery in the management of early breast cancer. Breast. 2017;34(Suppl 1):S1‐S4. 28673537 10.1016/j.breast.2017.06.018

[9] Fisher B . Laboratory and clinical research in breast cancer: a personal adventure—the David a Karnofsky memorial lecture. Cancer Res. 1980;40(11):3863‐3874. 7008932

[10] von Minckwitz G , Procter M , de Azambuja E , et al. Adjuvant pertuzumab and trastuzumab in early HER2‐positive breast cancer. N Engl J Med. 2017;377(2):122‐131. 28581356 10.1056/NEJMoa1703643 PMC5538020

[11] Piccart M , Procter M , Fumagalli D , et al. Adjuvant pertuzumab and trastuzumab in early HER2‐positive breast cancer in the APHINITY trial: 6 Years' follow‐up. J Clin Oncol. 2021;39(13):1448‐1457. 33539215 10.1200/JCO.20.01204

[12] Johnston SRD , Harbeck N , Hegg R , et al. monarchE committee members and investigators. Abemaciclib combined with endocrine therapy for the adjuvant treatment of HR+, HER2‐, node‐positive, high‐risk, early breast cancer (monarchE). J Clin Oncol. 2020;38(34):3987‐3998. 32954927 10.1200/JCO.20.02514 PMC7768339

[13] Schmid P , Cortes J , Pusztai L , et al. KEYNOTE‐522 investigators. Pembrolizumab for early triple‐negative breast cancer. N Engl J Med. 2020;382(9):810‐821. 32101663 10.1056/NEJMoa1910549

[14] Vanni G , Materazzo M , Perretta T , et al. Impact of awake breast cancer surgery on postoperative lymphocyte responses. In Vivo. 2019;33(6):1879‐1884. 31662515 10.21873/invivo.11681 PMC6899130

[15] Kim R , Kawai A , Wakisaka M , et al. Outcomes of outpatient breast cancer surgery at a private breast clinic. Breast J. 2018;24(4):628‐632. 29537724 10.1111/tbj.13012

[16] Kim R , Kawai A , Wakisaka M , et al. Outpatient breast‐conserving surgery for breast cancer: use of local and intravenous anesthesia and/or sedation may reduce recurrence and improve survival. Ann Med Surg (Lond). 2020;60:365‐371. 33224492 10.1016/j.amsu.2020.10.072 PMC7666315

[17] Melamed R , Bar‐Yosef S , Shakhar G , Shakhar K , Ben‐Eliyahu S . Suppression of natural killer cell activity and promotion of tumor metastasis by ketamine, thiopental, and halothane, but not by propofol: mediating mechanisms and prophylactic measures. Anesth Analg. 2003;97(5):1331‐1339. 14570648 10.1213/01.ANE.0000082995.44040.07

[18] Kushida A , Inada T , Shingu K . Enhancement of antitumor immunity after propofol treatment in mice. Immunopharmacol Immunotoxicol. 2007;29(3–4):477‐486. 18075859 10.1080/08923970701675085

[19] Yardeni IZ , Beilin B , Mayburd E , Alcalay Y , Bessler H . Relationship between fentanyl dosage and immune function in the postoperative period. J Opioid Manag. 2008;4(1):27‐33. 18444445 10.5055/jom.2008.0005

[20] Chamaraux‐Tran TN , Mathelin C , Aprahamian M , et al. Antitumor effects of lidocaine on human breast cancer cells: an *in vitro* and *in vivo* experimental trial. Anticancer Res. 2018;38(1):95‐105. 29277761 10.21873/anticanres.12196

[21] Huang Y , Pan L , Helou K , et al. Mechanical ventilation promotes lung metastasis in experimental 4T1 breast cancer lung‐metastasized models. Cancer Manag Res. 2018;10:545‐555. 29593433 10.2147/CMAR.S142650 PMC5865578

[22] Greten FR , Grivennikov SI . Inflammation and cancer: triggers, mechanisms, and consequences. Immunity. 2019;51(1):27‐41. 31315034 10.1016/j.immuni.2019.06.025 PMC6831096

[23] Brierley JD , Gospodarowicz MK , Wittekind C . TNM Classification of Malignant Tumours. 8th ed. WILEY‐Blackwell; 2017.

[24] Osako T , Iwase T , Kimura K , et al. Intraoperative molecular assay for sentinel lymph node metastases in early stage breast cancer: a comparative analysis between one‐step nucleic acid amplification whole node assay and routine frozen section histology. Cancer. 2011;117(19):4365‐4374. 21437889 10.1002/cncr.26060

[25] Giuliano AE , Ballman K , McCall L , et al. Locoregional recurrence after sentinel lymph node dissection with or without axillary dissection in patients with sentinel lymph node metastases: long‐term follow‐up from the American College of Surgeons oncology group (Alliance) ACOSOG Z0011 randomized trial. Ann Surg. 2016;264(3):413‐420. 27513155 10.1097/SLA.0000000000001863 PMC5070540

[26] Kurosumi M , Akashi‐Tanaka S , Akiyama F , et al. Committee for Production of histopathological criteria for assessment of therapeutic response of Japanese breast cancer society. Histopathological criteria for assessment of therapeutic response in breast cancer (2007 version). Breast Cancer. 2008;15(1):5‐7. 18224386 10.1007/s12282-007-0016-x

[27] Horii R , Akiyama F . Histological assessment of therapeutic response in breast cancer. Breast Cancer. 2016;23(4):540‐545. 24173652 10.1007/s12282-013-0499-6

[28] van Maaren MC , de Munck L , Strobbe LJA , et al. Ten‐year recurrence rates for breast cancer subtypes in The Netherlands: a large population‐based study. Int J Cancer. 2019;144(2):263‐272. 30368776 10.1002/ijc.31914

[29] Stuart‐Harris R , Dahlstrom JE , Gupta R , Zhang Y , Craft P , Shadbolt B . Recurrence in early breast cancer: analysis of data from 3,765 Australian women treated between 1997 and 2015. Breast. 2019;44:153‐159. 30785024 10.1016/j.breast.2019.02.004

[30] Masuda N , Lee SJ , Ohtani S , et al. Adjuvant capecitabine for breast cancer after preoperative chemotherapy. N Engl J Med. 2017;376(22):2147‐2159. 28564564 10.1056/NEJMoa1612645

[31] Kim R , Kawai A , Wakisaka M , et al. Immune correlates of the differing pathological and therapeutic effects of neoadjuvant chemotherapy in breast cancer. Eur J Surg Oncol. 2020;46(1):77‐84. 31563296 10.1016/j.ejso.2019.09.146

[32] Kim R , Kawai A , Wakisaka M , et al. Immune factors associated with the pathological and therapeutic effects of preoperative chemotherapy in patients with breast cancer. Transl Oncol. 2021;14(1):100927. 33157515 10.1016/j.tranon.2020.100927 PMC7649526

[33] Wang YJ , Fletcher R , Yu J , Zhang L . Immunogenic effects of chemotherapy‐induced tumor cell death. Genes Dis. 2018;5(3):194‐203. 30320184 10.1016/j.gendis.2018.05.003 PMC6176216

[34] Hodge JW , Garnett CT , Farsaci B , et al. Chemotherapy‐induced immunogenic modulation of tumor cells enhances killing by cytotoxic T lymphocytes and is distinct from immunogenic cell death. Int J Cancer. 2013;133(3):624‐636. 23364915 10.1002/ijc.28070 PMC3663913

[35] Fumet JD , Limagne E , Thibaudin M , Ghiringhelli F . Immunogenic cell death and elimination of immunosuppressive cells: a double‐edged sword of chemotherapy. Cancers (Basel). 2020;12(9):2637. 32947882 10.3390/cancers12092637 PMC7565832

[36] Kim R , Kin T . Current and future therapies for immunogenic cell death and related molecules to potentially cure primary breast cancer. Cancers (Basel). 2021;13(19):4756. 34638242 10.3390/cancers13194756 PMC8507525

[37] Ghiringhelli F , Apetoh L . Enhancing the anticancer effects of 5‐fluorouracil: current challenges and future perspectives. Biom J. 2015;38(2):111‐116. 10.4103/2319-4170.130923 25163503

[38] Bracci L , Schiavoni G , Sistigu A , Belardelli F . Immune‐based mechanisms of cytotoxic chemotherapy: implications for the design of novel and rationale‐based combined treatments against cancer. Cell Death Differ. 2014;21(1):15‐25. 23787994 10.1038/cdd.2013.67 PMC3857622

[39] Asleh K , Brauer HA , Sullivan A , et al. Predictive biomarkers for adjuvant capecitabine benefit in early‐stage triple‐negative breast cancer in the FinXX clinical trial. Clin Cancer Res. 2020;26(11):2603‐2614. 32005747 10.1158/1078-0432.CCR-19-1945

[40] Huang B , Yan X , Li Y . Cancer stem cell for tumor therapy. Cancers (Basel). 2021;13(19):4814. 34638298 10.3390/cancers13194814 PMC8508418

[41] Sauer S , Reed DR , Ihnat M , Hurst RE , Warshawsky D , Barkan D . Innovative approaches in the battle against cancer recurrence: novel strategies to combat dormant disseminated tumor cells. Front Oncol. 2021;11:659963. 33987095 10.3389/fonc.2021.659963 PMC8111294

[42] Walker ND , Elias M , Guiro K , et al. Exosomes from differentially activated macrophages influence dormancy or resurgence of breast cancer cells within bone marrow stroma. Cell Death Dis. 2019;10(2):59. 30683851 10.1038/s41419-019-1304-z PMC6347644

[43] De Angelis ML , Francescangeli F , Zeuner A . Breast cancer stem cells as drivers of tumor chemoresistance, dormancy and relapse: new challenges and therapeutic opportunities. Cancers (Basel). 2019;11(10):1569. 31619007 10.3390/cancers11101569 PMC6826533

[44] Karmakar MK , Samy W , Lee A , et al. Survival analysis of patients with breast cancer undergoing a modified radical mastectomy with or without a thoracic paravertebral block: a 5‐year follow‐up of a randomized controlled trial. Anticancer Res. 2017;37(10):5813‐5820. 28982906 10.21873/anticanres.12024

[45] Sessler DI , Pei L , Huang Y , et al. Breast cancer recurrence collaboration. Recurrence of breast cancer after regional or general anaesthesia: a randomised controlled trial. Lancet. 2019;394(10211):1807‐1815. 31645288 10.1016/S0140-6736(19)32313-X

[46] Kim R , Kawai A , Wakisaka M , Kin T . Current status and prospects of anesthesia and breast cancer: does anesthetic technique affect recurrence and survival rates in breast cancer surgery? Front Oncol. 2022;12:795864. 35223475 10.3389/fonc.2022.795864 PMC8864113

